# Dissolution of Silver Nanowires and Nanospheres Dictates Their Toxicity to *Escherichia coli*

**DOI:** 10.1155/2013/819252

**Published:** 2013-08-20

**Authors:** Meeri Visnapuu, Urmas Joost, Katre Juganson, Kai Künnis-Beres, Anne Kahru, Vambola Kisand, Angela Ivask

**Affiliations:** ^1^Institute of Physics, University of Tartu, Riia 142, 51014 Tartu, Estonia; ^2^Estonian Nanotechnology Competence Center, Riia 142, 51014 Tartu, Estonia; ^3^Laboratory of Environmental Toxicology, National Institute of Chemical Physics and Biophysics, Akadeemia 23, 12618 Tallinn, Estonia; ^4^Department of Chemistry, Tallinn University of Technology, Akadeemia 15, 12618 Tallinn, Estonia

## Abstract

Silver nanoparticles are extensively used in antibacterial applications. However, the mechanisms of their antibacterial action are not yet fully explored. We studied the solubility-driven toxicity of 100 × 6100 nm (mean primary diameter × length) silver nanowires (NWs) to recombinant bioluminescent *Escherichia coli* as a target representative of enteric pathogens. The bacteria were exposed to silver nanostructures in water to exclude the speciation-driven alterations. Spherical silver nanoparticles (83 nm mean primary size) were used as a control for the effect of NPs shape. Toxicity of both Ag NWs and spheres to *E. coli* was observed at similar nominal concentrations: the 4h EC50 values, calculated on the basis of inhibition of bacterial bioluminescence, were 0.42 ± 0.06 and 0.68 ± 0.01 mg Ag/L, respectively. Dissolution and bioavailability of Ag from NWs and nanospheres, analyzed with AAS or Ag-sensor bacteria, respectively, suggested that the toxic effects were caused by solubilized Ag^+^ ions. Moreover, the antibacterial activities of Ag NWs suspension and its ultracentrifuged particle-free supernatant were equal. The latter indicated that the toxic effects of ~80–100 nm Ag nanostructures to *Escherichia coli* were solely dependent on their dissolution and no shape-induced/related effects were observed. Yet, additional nanospecific effects could come into play in case of smaller nanosilver particles.

## 1. Introduction

Nanoparticles are included in various consumer goods. The most widely used are silver nanoparticles (Ag NPs) which, due to their antimicrobial effects [1, 2], are being incorporated into medical equipment coatings, cosmetic products, textiles, sprays, and other products (http://www.nanotechproject.org/). In addition to antimicrobial activity, Ag NPs possess characteristics that enable their use in electronics [3], solar energy conversion [4], or biosensing [5]. Current synthesis methods enable the production of differently shaped and sized Ag NPs. While mostly spherical Ag nanoparticles are produced, Ag nanowires have found increasing applications in different technologies, for example, in circuits to be used in nanoelectronic applications [6] or in solar cells [4].

The widespread use of Ag NPs has raised a concern about their environmental release and the subsequent effects. It has been clearly shown that studies on ecotoxicological effects of nanomaterials, including silver, drastically lag behind the design of new nanomaterials [7]. From the current studies it is clear that Ag NPs exhibit toxic effects to key organisms of the aquatic environment [8, 9]. These effects have mostly been explained by a combination of dissolved Ag^+^ ions and specific nanoeffects [10]. In case of bacteria, toxicity of Ag NPs has been shown to be driven by (i) attachment to the surface of the cell membrane and the following disturbance of membrane permeability and respiration [11], (ii) penetration of the cell membrane of the bacteria (1–10 nm Ag particles) and induction of subsequent physiological effects [12], and (iii) release of silver ions [13–15]. Although the released silver ions have been considered the main drivers for Ag NPs' antibacterial effects, it has been suggested that the extent of dissolution depends on nanoparticles' size and surface area as well as on the shape. Despite a large number of toxicological and ecotoxicological studies available for Ag NPs, most of these have been conducted using spherical particles. Only a few studies have explored the toxic effects of distinct shapes of nanosized Ag. Pal et al. [16] showed that among spherical, truncated triangular (both ~40 nm in diameter) and rod-shaped (~100 nm in length and 10 nm in diameter) Ag NPs the triangular Ag nanoplates had the most pronounced antibacterial effects. They suggested that the truncated particles exhibited higher area of active facets with {111} lattice plane which has high biological reactivity. In similar line, Sadeghi et al. [17] reported that among differentially shaped monodisperse Ag nanoparticles, nanoplates exhibited the highest toxicity to *Escherichia coli* and *Streptococcus mutans*. The same study also showed that Ag nanorods were more toxic to bacteria than Ag nanospheres. Yet, as particle sizes were not reported in this study, no conclusion on whether the differential toxicity was indeed caused by different shapes of Ag nanoparticles or their different sizes could be drawn. Another study by Ashkarran et al. [18] showed that spherical (17 nm hydrodynamic size) and rod-shaped (92 nm hydrodynamic size) Ag particles had similar toxicity to *E. coli* and *Bacillus subtilis*, while triangular Ag plates (50 nm hydrodynamic size) were less toxic. Shape-dependent toxicity of nanoparticles to bacterial cells has also been reported for other NPs than silver. For example, Liu et al. [19] reported that rod-shaped carbon structures exhibited antibacterial activity as these particles punctured bacterial cells. Thus, in general, the currently available studies indicate that the toxic effects of Ag nanoparticles may be increased by changing the shape of the particles from spherical to wires or triangles. This suggestion is supported by the few existing shape-related toxicity studies on cell cultures. Changing the shape of Ag particles from spherical to triangular and rod-shape was shown to increase the toxicity of Ag NPs to fish gill epithelial cells and zebrafish embryos [20] and to human alveolar epithelial cells [21]. Thus, it could be hypothesized that the rod/wire-shaped Ag nanoparticles could integrate the antimicrobial effects of silver with the inherent toxicity of nanosized wires. To test that hypothesis, we studied the antibacterial effects of silver nanowires (100 nm in diameter and 6100 nm in length) to a bioluminescent *Escherichia coli *strain as a surrogate or representative of closely related enteric opportunistic pathogens in humans. As a control for the shape, silver nanospheres with similar particle diameter (83 nm) were tested in parallel. The contribution of Ag^+^ ions to overall toxicity was evaluated by determining the dissolution of the studied Ag NPs using atomic absorption spectroscopy (AAS). From the results it was evident that the toxicity and bioavailability of both wire-shaped and spherical nanoparticles to *E. coli* were dependent on nanoparticles dissolution. Although particle dissolution explained the toxic effects of the relatively large Ag nanowires used in this study, smaller nanowires may also exhibit shape-related cellular effects.

## 2. Materials and Methods

### 2.1. Materials

Water purified with MilliQ equipment (Millipore, USA) and reagent grade chemicals were used throughout the experiments. Components of bacterial growth media were purchased from LabM (UK). Ampicillin sodium salt and tetracycline hydrochloride were purchased from Sigma-Aldrich. AgNO_3_ (used as the positive control for Ag^+^ ions due to its 100% dissolution in water) was purchased from J.T Baker (USA). 0.05 M stock solution in water was used for toxicological experiments. Ag nanowires (NWs) were purchased from Seashell Technology (USA) as suspensions in isopropanol. Before the test, Ag NWs were dried and redispersed in water at 5000 mg/L using Digital Sonifier 450 (Branson Ultrashell, Germany) probe sonicator at 40 W for 1.5 min. Ag nanospheres (83 nm average primary size; citrate stabilized) were purchased as aqueous suspensions (72 mg/L) from MKNano (Canada). Before the test, the stock suspension of Ag nanospheres was sonicated as described above but for 2.5 min. Both Ag NWs and Ag nanospheres were diluted in water to the desired concentration. Exact concentrations of Ag NWs and spheres in the tests were determined using GF-AAS in a certified laboratory.

### 2.2. Nanoparticle Characterization

Electron spectroscopic images from Ag particles were taken using SEM-FIB-EDX instrument (FEI Helios NanoLab 600, USA). SEM samples were prepared by depositing drops of aqueous suspensions of Ag nanoparticles on a silicon wafer. The samples were allowed to dry overnight at room temperature. The images were taken using an electron beam energy corresponding to 15 kV high voltage. 20 particles were measured from images using image processing software ImageJ (http://rsbweb.nih.gov/ij/) to obtain the primary dimensions of the nanostructures. Elemental analysis of the particles was performed using energy-dispersive X-ray spectroscopy (EDX) function (Oxford Instruments, UK) of the FIB-SEM-EDX setup. The EDX measurements were conducted by using primary electron beam with an acceleration voltage of 15 kV to detect Ag L*α* X-ray fluorescence (2.98 keV) [22]. The hydrodynamic diameter and surface charge (zeta potential) of Ag nanospheres (15 mg/L) and Ag nanowires (17 mg/L) were measured using Zetasizer Nano ZS (Malvern Instruments, UK) using dynamic light scattering (DLS) and electrophoretic light scattering (ELS) functions, respectively. DLS results are applicable for spherical particles only (User manual from Malvern Instruments) and thus were not used for Ag NWs. ELS results were used for Ag nanospheres as well as for Ag NWs. UV-Vis absorption spectra of Ag NWs (100 mg/L) and Ag nanospheres (10 mg/L) aqueous suspensions were recorded using a dual-beam spectrophotometer Multiskan Spectrum (Thermo Scientific) in 300–600 nm wavelength range as suggested by Chen et al. [23], Evanoff and Chumanov [24], and Yang et al. [25]. pH of the Ag NWs and Ag spheres suspensions was measured with pH meter Orion 8220 BNWP (Thermo Scientific, USA).

Dissolution of Ag^+^ ions from Ag nanostructures was analyzed in the suspensions equal to the highest concentration used in toxicological tests: 36 mg of Ag spheres/L and 15 mg Ag NWs/L. After preparation, the suspensions were incubated at room temperature for 4 h (the time duration also used for the bacterial toxicity assay) and then ultracentrifuged at 390 000 g for 45 minutes as suggested by Bondarenko et al. [15] and Ma et al. [26], to separate the particulate and ionic fraction. The carefully removed supernatant was analyzed for Ag using GF-AAS in a certified laboratory. Part of the supernatant was also used for the toxicity assay as described below.

### 2.3. Toxicity and Bioavailability Tests Using Recombinant *Escherichia***** *coli*

Two recombinant bioluminescent *Escherichia coli* strains were used (Table 1): *toxicity* was analyzed using constitutively bioluminescent bacterium *E. coli* MC1061(pSLlux) and *bioavailability of silver ions (Ag* ^+^ *) released from Ag nanostructures* was measured using a Ag^+^-induced bacterium *E. coli* MC1061(pSLcueR/pDNcopAlux).

**Figure 1:**
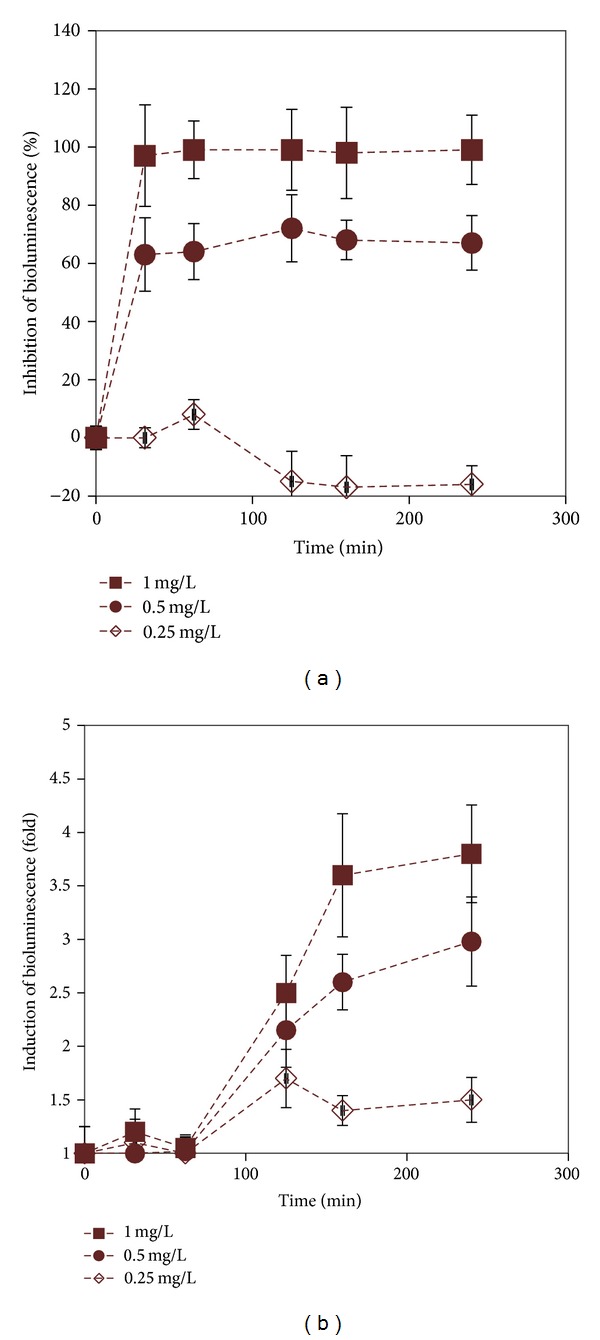
(a) Inhibition of bioluminescence (% of the control) in bioluminescent* Escherichia coli* in time and (b) induction of bioluminescence in Ag^+^-induced *Escherichia coli* in time upon exposure to different nominal concentrations of Ag nanowires (concentrations shown on the graphs). Note that the significant induction of Ag^+^-induced *E. coli* was observed only since 120 min of exposure and the induction increased with time. 240 min incubation was chosen for further experiments as this time resulted in highly significant induction, and the signal was easily distinguishable from the background (not induced) bioluminescence. Data represent mean ± standard deviation of three experiments. Negative inhibition values in (a) indicate small increase of bacterial bioluminescence at relatively low concentrations of Ag NWs, likely due to the hormesis effect [28].

Before the test, the bacteria were cultivated in LB medium (per litre: 10 g tryptone, 5 g of yeast extract and NaCl) supplemented with 100 *μ*g/mL of ampicillin (bioluminescent* E. coli*) or with 100 *μ*g/mL of ampicillin and 10 *μ*g/mL of tetracycline (Ag^+^-induced* E. coli*) to OD600 of 0.6. Then, the cells were washed twice with water by repeating the following cycle: centrifugation of the cells at 5000 g, removing the supernatant and adding similar volume of water. For toxicity and bioavailability assays, the OD600 of the bacterial culture was adjusted with water to 0.2 (bioluminescent* E. coli*) or 0.4 (Ag^+^-induced* E. coli*). 100 *μ*L of serially diluted aqueous suspensions of Ag nanowires, Ag nanospheres, their ultracentrifuged supernatants, and AgNO_3_ (solubility control for both nanostructures) were transferred to white microplate (polystyrene, Greiner) wells, each concentration in two replicates. Water was used as the negative control. Then, 100 *μ*L of bacterial suspension was added to each well and the plate was incubated in the dark at room temperature (about 20°C). Bioluminescence of the bacteria was registered at least every hour during 4 hours using Microplate Luminometer Orion II (Berthold Detection Systems). The 4-hour incubation time was chosen as a compromise: during that relatively long exposure period significant induction of the luminescence of the Ag^+^-induced* E. coli* was observed (Figure 1(b)) yet no effect on the sensitivity of the constitutively luminescent bioluminescent* E. coli* was recorded (Figure 1(a)). All the experiments were carried out over two or three separate days in order to take into account the inherent variability of the bioassays.

Inhibition of bioluminescence of bioluminescent* E. coli* was calculated by the following formula:

(1) $$\begin{array}{c} \text{Inhibition}\;\left(\%\right)=100-\frac{{\text{RLU}}_{S}}{{\text{RLU}}_{B}}\times 100, \end{array}$$

where RLU_*S*_ is bacterial bioluminescence in the sample and RLU_*B*_ is bacterial bioluminescence in water. EC50 was calculated by plotting the log_10_ values of Ag concentrations against bioluminescence inhibition using GraphPad program. 

Induction of the Ag^+^-induced *E. coli* was calculated by the following formula:

(2) $$\begin{array}{c} \text{Induction}\;\left(\text{fold}\right)=\frac{{\text{RLU}}_{S}}{{\text{RLU}}_{B}}. \end{array}$$

Induction value of 2 was considered as induction threshold as suggested by Hakkila et al. [29]. Bioavailability of Ag to sensor bacteria was calculated from induction threshold. Both EC50 and bioavailability values were normalized to dissolved Ag, determined from the ultracentrifuged extracts of Ag nanostructures by GF-AAS. 

In addition to bioluminescence inhibition, the impact of Ag nanostructures on bacterial viability was also evaluated. Once the bacteria had been exposed to Ag NWs for 4 hours for bioluminescence measurement, 2 *μ*L of the bacteria-sample mixture was pipetted onto LB agar plates and the plates were incubated at 30°C for 24 hours. The potential of bacterial cells to form colonies on LB plates after being exposed to AgNWs for 4 hours was visually evaluated.

### 2.4. Statistical Analysis

95% confidence intervals of EC50 and induction values were calculated using the program GraphPad. MS Excel was used to calculate standard deviations and perform *t*-test.

## 3. Results and Discussion

### 3.1. Characterization of Ag Nanowires and Spheres

**Figure 2:**
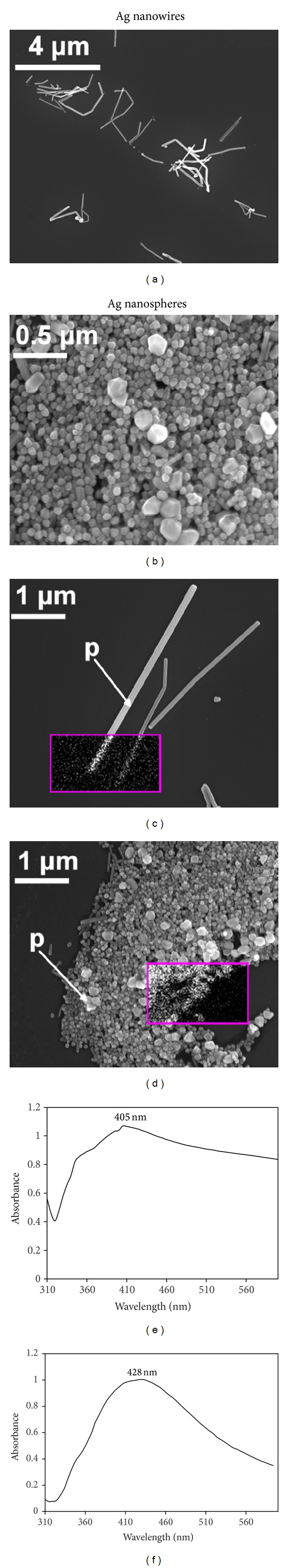
Scanning electron micrographs (SEM), EDX mapping, and UV-vis spectra of the Ag nanowires (NWs) and Ag nanospheres. (a) and (c) SEM micrographs of Ag NWs, (b) and (d) SEM micrographs of Ag nanospheres; note different magnifications; scale bars are indicated. Insets in (c) and (d) show EDX mapping. (e) UV-vis spectrum of Ag NWs and (f) UV-vis spectrum of Ag nanospheres. Maximum absorbance values are shown in (e) and (f).

Electron micrographs of Ag nanowires (Ag NWs) and spherical particles that were used as control nanoparticles (NPs) are shown in Figure 2. EDX point analysis made to the regions indicated with the letter “p” in Figure 2 confirmed that particles only consisted of Ag. Ag NWs were 70–150 nm in diameter and 3000–8000 nm in length (Table 2). The diameter of Ag spheres according to SEM measurements was 50–100 nm. The average hydrodynamic diameter of Ag spheres in water, based on DLS analysis, was 98 nm (Table 2). EDX mapping images (Figure 2) indicate that the observed nanostructures only contained Ag in addition to Si and C signals that originated from the underlying sample holder. UV-vis spectra of Ag NWs and spheres are shown in Figure 2. As expected, the absorption peak of spherical particles was narrower than that of NWs. Similar result was shown earlier by Ashkarran et al. [18]. Absorption peak of Ag NWs at 380 nm corresponds to transverse plasmon mode of Ag NWs, and the shoulder peak at ~355 nm can be attributed to long nanowires which exhibit similar optical properties to bulk silver [30].

The surface charge of both nanostructures was highly negative (−36 and −46 mV) (Table 2). Also, both nanostructures dissolved at similar rate: 2.4% of Ag NWs and 2.2% of Ag spheres were in the form of Ag^+^ ions in the aqueous suspensions of these nanostructures (Table 2).

The relatively similar primary particle diameter and negative surface charge as well as similar dissolution rate (Table 2) supported the use of Ag nanospheres as controls for Ag NWs shape in further experiments.

### 3.2. Toxicity and Bioavailability of Ag Nanowires to *Escherichia coli*

**Figure 3:**
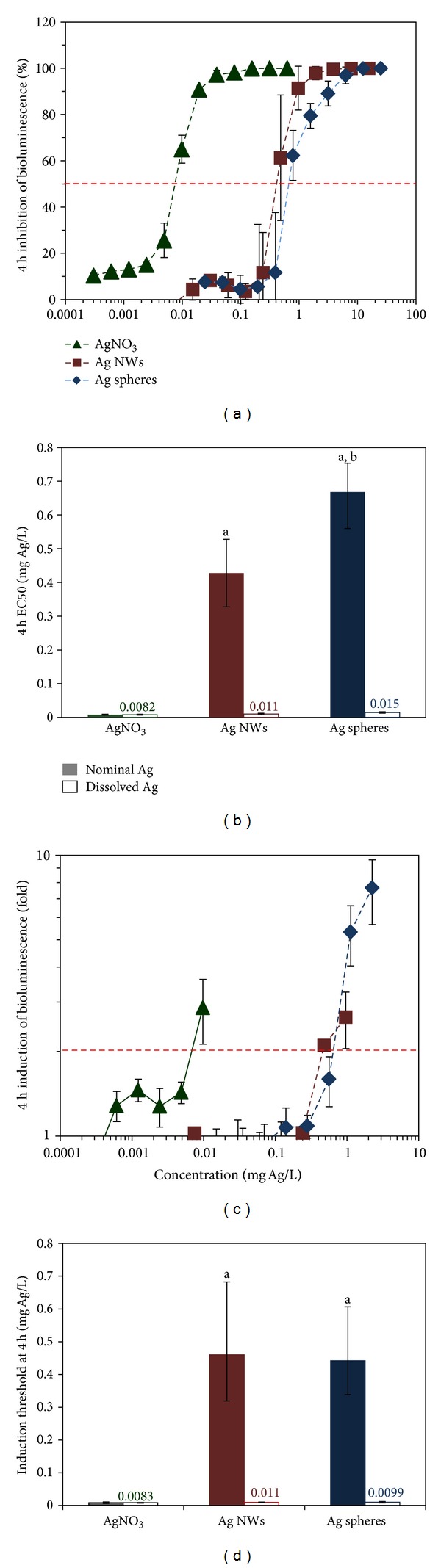
Dose-dependent effects of Ag nanowires (NWs), Ag nanospheres, and AgNO_3_ on two recombinant *Escherichia coli* strains after 4h exposure. (a) Inhibition of the light output in bioluminescent *E. coli*; (c) induction of the bioluminescence of the Ag^+^-induced *E. coli*. Filled columns in (b) indicate EC50 values calculated from panel (a) (see horizontal dotted line); filled columns in (d) indicate induction threshold values calculated from panel (c) (see horizontal dotted line). Open columns in (b) and (d) with numerical values show mg of dissolved Ag/L at EC50 or induction threshold, respectively. Mean ± standard deviation of two independent experiments is shown: a—significant (*P* < 0.05) difference from AgNO_3_, b—significant (*P* < 0.05) difference from AgNO_3_ and Ag NW.

Inhibition of light output of bioluminescent *E. coli* in response to its exposure to Ag nanostructures and comparatively to AgNO_3_ as a solubility control is shown in Figure 3(a). The respective EC50 values are indicated in Figure 3(b). The EC50 values of Ag NWs and spherical Ag nanoparticles were 0.42 ± 0.06 and 0.68 ± 0.01 mg Ag/L, respectively (Figure 3). In parallel experiments the EC50 value for AgNO_3_ (control for dissolved Ag^+^ ions) was 0.0082 mg/L. Taking into account the dissolution of Ag NWs and spheres (Table 2), the calculated amount of dissolved Ag^+^ ions at EC50 concentrations of Ag NWs and spheres was 0.011 and 0.015 mg/L, respectively (Figure 3(b), open columns). These values were not statistically significantly different from the EC50 value of AgNO_3_ and thus, suggest that the observed toxic effects of Ag NWs and spheres were driven by dissolved Ag^+^ ions. Further evidence that Ag NWs and spheres exhibited their toxicity *via* dissolved metal ions was obtained using Ag^+^-induced bacterial cells. When exposed to Ag NWs and spheres, the Ag^+^-induced bacteria were induced by similar nominal concentrations, 0.46 ± 0.2 and 0.44 ± 0.16 mg Ag/L, respectively (Figures 3(c) and 3(d)) indicating that similar amount of Ag^+^ ions was released from Ag nanoparticles and entered bacterial cells. Again, when the nominal inducing concentrations of Ag NWs and spheres were corrected for dissolution, the dissolved concentration of Ag in the induction threshold of the Ag^+^-induced bacteria was statistically similar in case of all Ag formulations (Figure 3(d)).

**Figure 4:**
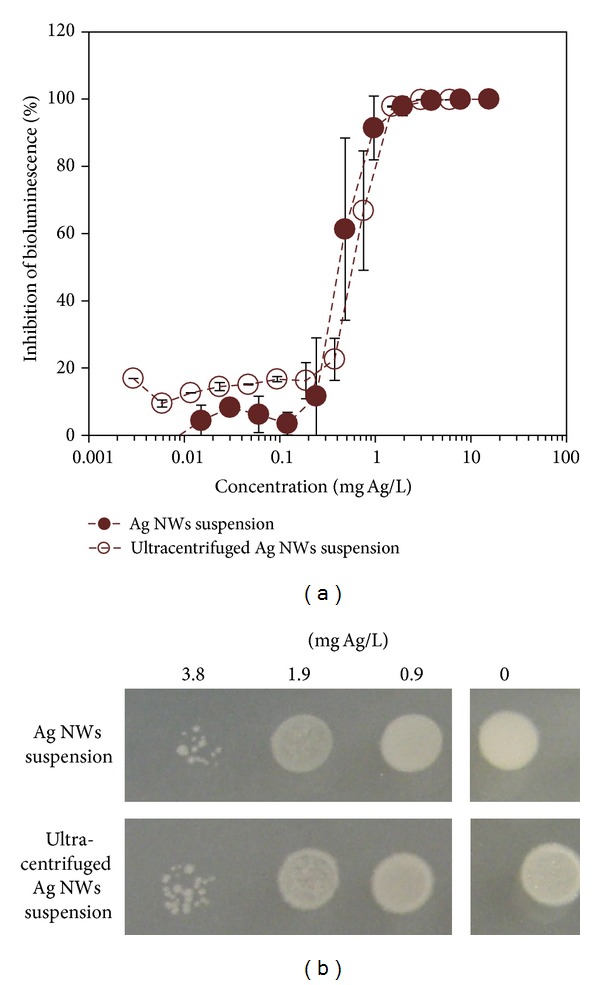
Antibacterial activity of Ag NWs and their particle-free supernatant to recombinant *Escherichia coli*. (a) Inhibition of bacterial bioluminescence by Ag NWs suspension (see also Figure 3(a)) and its supernatant (see Section 2). Mean ± standard deviation of three independent experiments is shown; (b) potential of *Escherichia coli* to yield colonies on agarized LB growth medium after 4-hour incubation with different nominal concentrations of Ag NWs suspension and the respective particle-free supernatant. Photo is taken after 24 h incubation of the inoculated test plates at 30°C. Opaque “spot” in (b) indicates high number of bacteria; appearance of individual colonies within the “spot” indicates decreased number of viable cells. All the Ag concentrations are nominal concentrations in Ag NWs suspension.

Additional evidence that dissolved Ag^+^ ions were the only cause of toxicity for Ag nanoparticles of this study was obtained by comparing the toxicity of Ag NWs dispersion and its ultracentrifuged extract (Figure 4). According to bioluminescence inhibition assay, the EC50 values of noncentrifuged Ag NWs dispersion and its ultracentrifuged extract did not statistically significantly differ being 0.42 ± 0.2 and 0.75 ± 0.25 mg Ag/L, respectively. Similarly, no visually significant differences were observed when the decrease in bacterial cell count by Ag NWs suspension or ultracentrifuged extract was tested. In both cases remarkable decrease in bacterial cell count was observed at 3.8 mg/L (Figure 4(b)). Thus, both bioluminescence inhibition assay and viability assay indicated that the removal of Ag NWs from the test did not decrease the NWs toxicity and hence suggested that toxicity of Ag NWs was indeed resulting from dissolved Ag^+^ ions.

Comparison of EC50 values from bioluminescence inhibition assay and concentrations of Ag NWs that decreased the bacterial viability showed that the inhibition of bacterial bioluminescence occurred at 5–9-fold lower concentrations than were the concentrations that affected cellular viability. That is an additional proof that the inhibition of bacterial bioluminescence, either natural luminescent bacteria *Vibrio fischeri* or recombinant luminescent *Escherichia coli,* is an early indicator for toxicity that reflects the changes in bacterial energy metabolism [31] at a stage where viability is not yet affected. Indeed, bacterial bioluminescence has been shown to very significantly correlate with cellular viability [32], justifying its application in toxicity testing.

In summary, the results from this study indicate that the toxicity of 100 × 6100 nm Ag nanowires and Ag nanospheres with particle diameter of 83 nm occurred in very similar concentration range and was dictated solely by dissolved Ag^+^ ions. This finding is in agreement with the recent papers by Xiu et al. [14] and Bondarenko et al. [15] who also suggested that dissolution is the key element determining the toxicity of Ag nanostructures. On the other hand, according to our initial hypothesis and earlier papers by Pal et al. [16], Sadeghi et al. [17], and Ashkarran et al. [18], the shape of Ag nanostructures was also expected to play a role in Ag NPs antibacterial effects and toxicity. Thus, the fact that no shape-dependent toxicity was observed for nanowires and spheres was somewhat surprising. On the other hand, in previous studies reporting on shape-dependent toxicity of Ag nanoparticles comparisons were performed between particles that had relatively different particle size. For example, the diameter of Ag nanorods (*∼*10 nm) in the study by Pal et al. [16] was remarkably smaller than the size of Ag triangles (*∼*40 nm) that were studied. Also, the diameters of Ag spheres, triangles, wires, and cubes used by Ashkarran et al. [18] ranged from 17 to 92 nm. Thus, in addition to specific effects of particle shape, the variable particle size may have also played a role in the differential toxicity of these particles. Furthermore, similarly to our study, no marked differences in antibacterial effects of Ag nanoparticles with slightly different shapes were reported by Sathishkumar et al. [33, 34]. Yet, it should be emphasized that the nanoparticles in our study were relatively large and our conclusion that antibacterial effects of Ag NPs do not depend on particles shape may not hold true in case of smaller nanoparticles. Clarification of this aspect requires further studies.

## 4. Conclusions and Outlook

In this study we showed that the toxicity of 100 × 6100 nm (diameter × length, mean primary size) Ag nanowires and 83 nm nanospheres (mean primary size) to gram-negative bacterium *E. coli*—a representative of enteric pathogens—was driven by dissolved Ag^+^ ions. Therefore, by regulating the solubilization of silver nanostructures, for example, by applying different coatings, it is possible to fine-tune the nanostructures towards higher toxicity, that is, to yield more efficient silver-based antibacterial agents, or, on the contrary, to render them less soluble if the main goal is other than antibacterial application (safe by design approach) [35].

Finally, although dissolution was shown to drive the toxicity of relatively large nanostructures as those studied in this paper, the possibility that the toxicity of smaller nanoparticles could also be driven by direct effects between nanoparticles and cells, that is, by “nano-specific” effects, cannot be excluded.

## Figures and Tables

**Table 1:** Characteristics of the applied *Escherichia coli* strains.

	Characteristics	Designation in the current study	Reference
*E. coli* MC1061 (pSLlux)	Constitutively bioluminescent; bioluminescence is decreased in response to interference in cellular energy production, effects on membranes, or decreased viability	Bioluminescent *E. coli *	[27]
*E. coli* MC1061 (pSLcueR/pDNcopAlux)	Ag^+^-induced strain; bioluminescence is increased in response to intracellular Ag^+^ ions	Ag^+^-induced *E. coli *	[27]

**Table 2:** Physicochemical characteristics of Ag nanospheres and nanowires (NWs).

	Primary size^1^, nm	*ζ*-potential^2^, mV (pH)	Hydrodynamic size^3^, nm (pdi)	Dissolution^4^, %
Ag NWs	100 ± 40 × 6100 ± 2700	−46 (7.0)	Not relevant^5 ^	2.4
Ag spheres	83 ± 37	−36 (7.2)	98 ± 1.8 (0.25)	2.2

^1^Determined from scanning electron micrographs; *n* = 20.

^2^Analyzed using electrophoretic light scattering method. The data were analyzed using Smoluchowski approximation.

^3^Hydrodynamic size is based on dynamic light scattering (DLS) measurement. pdi: polydispersity index.

^4^% nanomaterial dissolved was analyzed from ultracentrifuged extracts of 36 mg/L (Ag spheres) or 15 mg/L (Ag nanowires) dispersions by GF-AAS.

^5^Dynamic light scattering (DLS) measurement is not relevant for rod-shaped particles.
